# Genetic interaction screen for severe neurodevelopmental disorders reveals a functional link between *Ube3a* and *Mef2* in *Drosophila melanogaster*

**DOI:** 10.1038/s41598-020-58182-5

**Published:** 2020-01-27

**Authors:** Jonas Straub, Anne Gregor, Tatjana Sauerer, Anna Fliedner, Laila Distel, Christine Suchy, Arif B. Ekici, Fulvia Ferrazzi, Christiane Zweier

**Affiliations:** Institute of Human Genetics, Friedrich-Alexander-Universität Erlangen-Nürnberg (FAU), 91054 Erlangen, Germany

**Keywords:** Genetic interaction, Medical genetics, Neurodevelopmental disorders, Molecular medicine

## Abstract

Neurodevelopmental disorders (NDDs) are clinically and genetically extremely heterogeneous with shared phenotypes often associated with genes from the same networks. Mutations in *TCF4, MEF2C, UBE3A, ZEB2* or *ATRX* cause phenotypically overlapping, syndromic forms of NDDs with severe intellectual disability, epilepsy and microcephaly. To characterize potential functional links between these genes/proteins, we screened for genetic interactions in *Drosophila melanogaster*. We induced ubiquitous or tissue specific knockdown or overexpression of each single orthologous gene (*Da*, *Mef2*, *Ube3a*, *Zfh1*, *XNP*) and in pairwise combinations. Subsequently, we assessed parameters such as lethality, wing and eye morphology, neuromuscular junction morphology, bang sensitivity and climbing behaviour in comparison between single and pairwise dosage manipulations. We found most stringent evidence for genetic interaction between *Ube3a* and *Mef2* as simultaneous dosage manipulation in different tissues including glia, wing and eye resulted in multiple phenotype modifications. We subsequently found evidence for physical interaction between UBE3A and MEF2C also in human cells. Systematic pairwise assessment of the *Drosophila* orthologues of five genes implicated in clinically overlapping, severe NDDs and subsequent confirmation in a human cell line revealed interactions between UBE3A/Ube3a and MEF2C/Mef2, thus contributing to the characterization of the underlying molecular commonalities.

## Introduction

Neurodevelopmental disorders (NDDs) are clinically and genetically extremely heterogeneous, and currently more than 1,000 genes have been implicated (SysID database^1^). Within the last years, phenotypically and biologically coherent modules within this large and heterogeneous group have been increasingly delineated, indicating that overlapping phenotypes are often caused by mutations in genes involved in the same molecular networks^1–5^. This has been demonstrated mainly for well-defined pathways or complexes such as the RAS-MAPK-pathway^4^ or the SWI/SNF chromatin remodelling complex^6^, but is less characterized for disease genes involved in a broader biological context or process such as transcriptional regulation. Identifying and characterizing such connections and correlations contributes to a better understanding of the complex mechanisms and pathomechanisms in neurodevelopment and its associated disorders.

**Figure 1:**
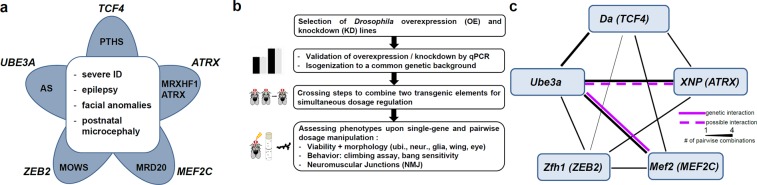
Genes of interest and study outline. (**a**) Five syndromic NDDs with considerable phenotypic overlap were selected. AS, Angelman syndrome; MOWS, Mowat-Wilson syndrome; MRD20, mental retardation, autosomal dominant 20 (MEF2C haploinsufficiency syndrome); MRHXF1, mental retardation-hypotonic facies syndrome/ATRX, alpha-thalassemia-mental retardation syndrome; PTHS, Pitt-Hopkins syndrome (**b**) Schematic drawing of the study outline and the work flow. (**c**) Overview of results from genetic interaction studies in *Drosophila*. Black lines indicate gene-gene connections investigated in this study by one (thin line), two (middle line) or four (thick line) different combinations. Magenta lines indicate genetic interactions between two genes, respectively. Genetic interaction was defined here as the observation of multiple phenotypic modifications upon pairwise dosage manipulation in several tissues. Solid magenta line indicates strongest evidence for genetic interaction with several consistent phenotypic modifications observed in different tissues. Dashed line in magenta indicates evidence for a possible genetic interaction with some phenotypic modifications observed. Single or inconsistent phenotypic modifications are not indicated.

Pitt-Hopkins syndrome (PTHS, MIM**#** 610954), *MEF2C*-related intellectual disability (MRD20; MIM**#** 613443), Mowat-Wilson syndrome (MOWS, MIM**#** 235730), *ATRX*-related intellectual disability (ATRX, MIM#301040; MRXHF1, MIM#309580) and Angelman syndrome (AS, MIM#105830) represent a particular group of syndromic NDDs. They are important differential diagnoses towards each other and share phenotypic characteristics such as severe intellectual disability, epilepsy, postnatal microcephaly and some facial features^7^ (Fig. 1a). Angelman syndrome is caused by loss of the maternal allele of *UBE3A*, encoding an ubiquitin-protein ligase^8,9^. PTHS, MRD20 and MOWS are caused by haploinsufficiency of *TCF4*, *MEF2C* or *ZEB2*, respectively, all encoding transcription factors^10–12^. Also X-chromosomal *ATRX*, implicated in *ATRX*-related intellectual disability, encodes a transcriptional regulator^13^.

*Drosophila melanogaster* has been demonstrated to be a powerful model to investigate functional relationships between NDD-associated genes/proteins, given a high conservation of genes, pathways and regulatory networks between flies and humans^14–17^. *Drosophila* screens for eye, wing and neuronal phenotypes upon RNAi-based knockdown of NDD-associated gene orthologues revealed robust correlations between fly and human phenotype groups^1,18^ in terms of phenologs^19^, thus indicating conservation of functional modules. More specific functional relationships between individual genes can be investigated by genetic interaction studies. Genetic interaction is defined as the observation that a double mutant’s phenotype deviates from what is expected from each individual mutant^20^. Such approaches, based on quantifiable phenotypes in *Drosophila*, were utilized to investigate multiple-hit and copy number variant (CNV) models as done e.g. for autism spectrum disorders^21,22^ or to validate newly identified NDD-associated candidate genes by establishing biological links to phenotypically overlapping, known NDD-associated genes in terms of a chromatin-modification module^5^.

By utilizing *Drosophila melanogaster* as a model to screen for genetic interactions, we identified specific functional links between several genes in the fly, most stringent between *Ube3a* and *Mef2*. This interaction was furthermore confirmed in a human cell line using co-immunoprecipitation experiments. These molecular commonalities might contribute to the clinically overlapping features of the investigated disorders.

## Results

### Ubiquitous and tissue specific dosage manipulation in *Drosophila melanogaster*

To systematically investigate functional links between these genes of interest, we used *Drosophila melanogaster* as an *in vivo* model system and tested genetic interaction between the fly orthologues Mef2 (MEF2C), Zfh1 (ZEB2), Daughterless (Da) (TCF4), XNP (ATRX) and Ube3a (UBE3A).

Quantitative reverse transcriptase PCR (RT-PCR) confirmed knockdown (KD) to 35–70% residual levels and 3 to 8.5fold overexpression (OE) for all used lines (Supplementary Table S1) except for *Zfh1* (Supplementary Fig. S1). Overexpression of *Zfh1* resulted in early lethality, preventing quantitative RT-PCR, and knockdown could not be shown (Supplementary Fig. S1), leading to exclusion of the KD_*Zfh1*-line from subsequent experiments. To evaluate the possibility of overlapping phenotypes resulting from dosage manipulation of *Ube3a*, *Mef2*, *Da, XNP* or *Zfh1* and to identify quantifiable phenotypes for subsequent genetic interaction experiments, we induced knockdown (four genes) or overexpression (five genes) either ubiquitously or in several different tissues and evaluated parameters such as viability, morphological alterations, synapse development and gross neurological behaviour (Fig. 1b).

**Figure 2:**
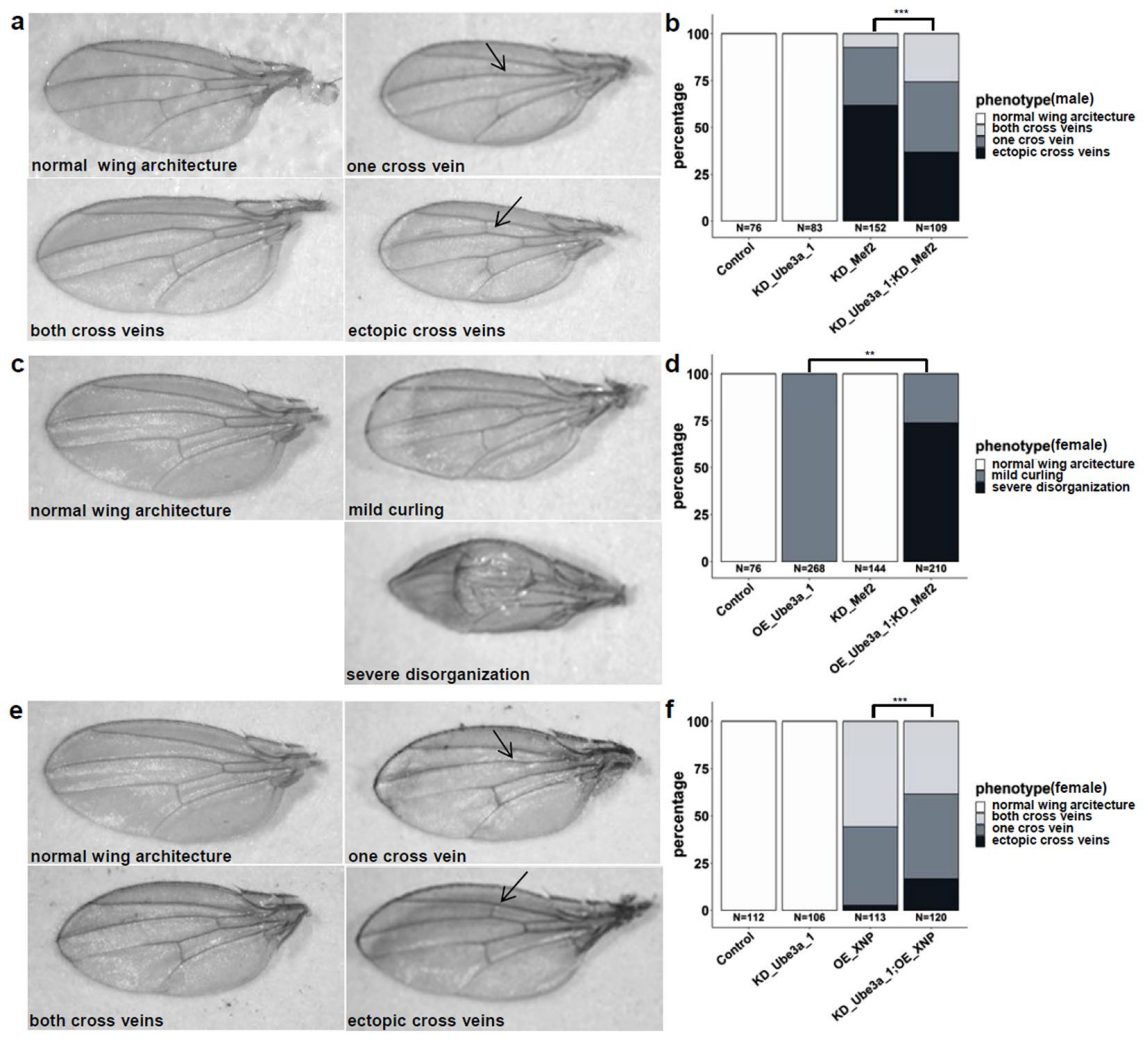
Genetic interaction of *Ube3a* and *Mef2* or *XNP* in the *Drosophila* wing (*ms1096*-GAL4). (**a**) Knockdown (KD) of *Ube3a* does not cause a wing phenotype, KD of *Mef2* causes abnormally curled wings in male flies with additional cross vein defects, such as missing anterior cross veins and/or ectopic cross veins (marked with an arrow). Simultaneous KD of *Ube3a* and *Mef2* results in a milder phenotype with significantly more flies with both cross veins present and fewer flies with ectopic cross veins, quantified in (**b**). (**c**) Overexpression (OE) of *Ube3a* causes abnormally curled wings in females, while KD of *Mef2* does not cause a phenotype in female flies (male phenotype see above). Simultaneous OE of *Ube3a* and KD of *Mef2* results in male lethality and in a more severe disorganization of wing architecture in about 75% of females, as quantified in (**d**). (**e**) OE of *XNP* causes abnormally curled wings in female flies with additional cross vein defects, such as missing anterior cross veins and/or ectopic cross veins (marked with an arrow). Simultaneous KD of *Ube3a* (normal) and OE of *XNP* results in a more severe phenotype with more flies with ectopic cross veins and fewer flies with both cross veins intact, as quantified in (**f**). Statistical analysis was performed using Fisher’s Exact test, **p ≤ 0.001; ***p ≤ 0.0001). Flies are counted towards the more severe phenotype if at least one wing was affected. These results are from an independent experiment than in Supplementary Table S2, thus numbers are different.

Ubiquitous knockdown of two of the four tested genes (*Mef2* and *Da*) and overexpression of all five tested genes resulted in lethality. Wing specific knockdown resulted in morphological phenotypes for two genes (*Mef2, Da*), and wing specific overexpression resulted in (male) lethality or morphological wing phenotypes for all five genes (Table 1, Figs. 2, S2).

**Table 1:** Viability and morphology upon tissue-specific dosage manipulation.

condition	ubi.	wing	eye	neur.	glia
**single knockdown**					
KD_*Ube3a*_1	N	N	N^18^	N	N
KD_*Ube3a*_2	N	N	N	N	N
KD_*Mef2*	L	P (curled, alt. veins)^62^	P (bubbles)	N	N
KD_*Da*	(L)	P^1^	P (red. bristles),^18^	N	N
KD_*XNP*	N	N^1^	N^18^	N	N
**single overexpression**					
OE_*Ube3a*_1	L	mL,P (curled)	P (rough)	(L)	N
OE_*Ube3a*_2	L	(mL),P	P (rough)	N	N
OE_*Mef2*	L	P^33^	P (red. bristles)	N	mL, (L)
OE_*Da*	L	mL,P	L	(mL)	L
OE_*XNP*	L	P (crumbled, curled, alt. veins)	N	N	L
OE_*Zfh1*	L	L	L	L	L
**pairwise manipulation**					
KD_*Ube3a*_1;KD_*Mef2*	L	P↑	P ↑	N	N
OE_*Ube3a*_1;OE_*Mef2*	L	mL, P↓	P ↓	(L)	L↓
KD_*Ube3a*_1;OE_*Mef2*	L	P	P	N	L↓
OE_*Ube3a*_1;KD_*Mef2*	L	mL,P↓	(mL)↓, P↓	L↓	L↓
KD_*Ube3a*;KD_*XNP*	N	N	N	N	N
OE_*Ube3a*_1;OE_*XNP*	L	mL,P	P	(L)	L
KD_*Ube3a*_1;OE_*XNP*	L	P↓	N	N	(L)
OE_*Ube3a*_1;KD_*XNP*	L	(mL)↑, P	P	mL ↓	N
KD_*Ube3a*_1; KD_*Da*	(L)	P	P	N	N
OE_*Da*;OE_*Ube3a*_2	L	mL,P	L	(mL)	L
OE_*Da*;KD_*Ube3a*_2	L	(L)↓	L	(mL)	L
OE_*Ube3a*_1;KD_*Da*	L	(mL),P	P	(L)↓	N
OE_*Da*;OE_*Mef2*	L	(L)↓	(L)	N(↑)	L
OE_*Da*;KD_*Mef2*	L	mL,P	L	(mL)	L
OE_*Da*;OE_*XNP*	L	mL,P	mL↑,P	(mL)	L
OE_*Da*;KD_*XNP*	L	mL,P	L	(mL)(↓)	L
OE _*Zfh1*;OE_*Ube3a*_2	L	L	L	L	L
OE_*Zfh1*;KD_*Ube3a*_2	(L)	L	L	L	L
OE_*Zfh1*;OE_*Mef2*	L	L	L	L	L
OE_*Zfh1*;KD_*Mef2*	L	L	L	L	L
OE_*Zfh1*;OE_*XNP*	L	L	L	L	L
OE _*Zfh1*;KD_*XNP*	L	L	L	L	L
OE_*Zfh1*;KD_*Da*	L	L	L	L	L

Ubi., ubiquitous; neur., pan-neuronal; N, normal viability and normal morphology; L, lethality; mL, male lethality; P, morphological phenotype; ↑, milder phenotype compared to single KD or OE; ↓, more severe phenotype compared to single KD or OE; (), incomplete penetrance or borderline phenotype/modification; some of the phenotypes have been reported previously for the same line or condition; References indicate reports with similar phenotypic observations (absence of phenotype or presence of identical phenotype) in previous studies.

**Figure 3:**
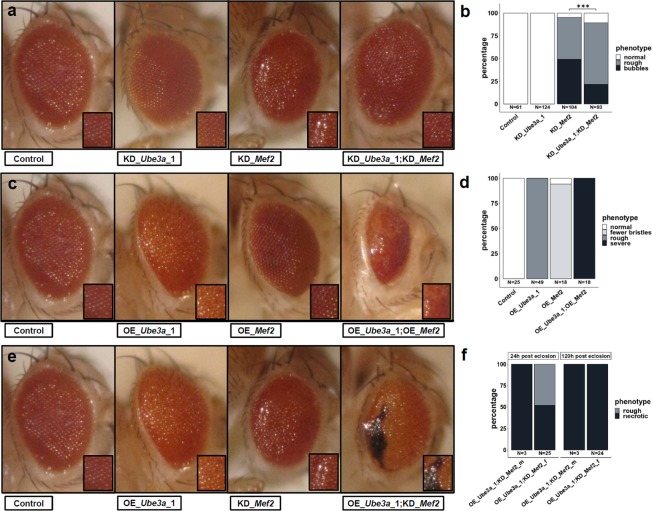
Genetic interaction of *Mef2* and *Ube3a* in the *Drosophila* eye (*GMR*-GAL4). (**a**) Knockdown (KD) of Ube3a does not cause a phenotype, KD of *Mef2* causes rough eyes with more severely affected flies also displaying a bubble-like appearance. Simultaneous KD of *Ube3a* and *Mef2* results in a milder phenotype with significantly fewer eyes with bubble-like appearance, quantified in (**b**) (***: p ≤ 0.001, Fisher’s Exact test). (**c**) Overexpression (OE) of *Ube3a* causes rough eyes, OE of *Mef2* results in a mildly reduced number of bristles but grossly intact ommatidial structure. Simultaneous OE of both results in a severe phenotype with reduced eye size and dissolved ommatidia structure in all eyes as quantified in (**d**). (**e**) Simultaneous OE of *Ube3a* (rough eye) and KD of *Mef2* (rough eyes and occasionally bubble-like appearance) results in a different and more severe phenotype with disorganized ommatidia structure and progressive necrosis as quantified for male and female flies in (**f**). Pictures and quantifications are from male flies if not indicated otherwise.

Eye-specific knockdown resulted in morphological phenotypes for two genes (*Mef2*, *Da*), and eye-specific overexpression resulted in lethality or morphological phenotypes for four of the genes (*Ube3a*, *Mef2*, *Da*, *Zfh1*) (Table 1, Figs. 3, S3).

Nervous system specific knockdown either pan-neuronally or in glia cells did not result in reduced viability. However, pan-neuronal overexpression of either *Zfh1*, *Da* or *Ube3a* or glial overexpression of either *Zfh1*, *Da*, *XNP* or *Mef2* resulted in variable lethality phenotypes (Tables 1 and S2). Evaluation of several parameters of larval neuromuscular junctions (NMJs) such as NMJ area, NMJ length, number of islands, branches, boutons or active zones did not reveal any consistent alterations in synaptic morphology upon either pan-neuronal knockdown or overexpression of any of the genes (Supplementary Fig. S4). We did not observe a satellite bouton phenotype (data not shown) as previously described in an *Ube3a* mutant^23^. This might be related to a weaker knockdown of the RNAi line. Furthermore, gross neurological functioning was tested using the climbing assay upon pan-neuronal knockdown or overexpression. We found that overexpression of *Da* or *XNP* resulted in a mild climbing defect, respectively, and that overexpression of *Ube3a* led to a severe climbing deficit (Supplementary Fig. S5). Additional testing of seizure susceptibility with the bang sensitivity assay (Supplementary Table S3) did not reveal consistent phenotypes for any of the tested KD and OE lines.

### Genetic interaction screen

Based on the location of RNAi- or UAS-elements on chromosomes 2 or 3, we could create three lines combining two KD elements each, eight lines combining two OE elements each, and twelve lines combining KD with OE elements each (Table 1, Fig. 1c). By quantitative RT-PCR we found no indication that distribution of GAL4 between two UAS-elements would dilute its effect (Supplementary Fig. S1). We tested all combinations for lethality and wing and eye morphology phenotypes. NMJ morphology, bang sensitivity and climbing behaviour were assessed for the three KD combination constructs and for the eight OE combinations.

Phenotypic modifications were considered as a) additive, when the combined phenotype represented the sum of the two single phenotypes, b) as antagonistic, when the combined phenotype was milder than the more severe of the two single phenotypes, and c) as synergistic, when the combined phenotype was different or more severe than what was expected from the sum of the two single phenotypes.

In the evaluation of viability and gross morphologic eye and wing phenotypes upon ubiquitous and tissues specific dosage manipulation we observed several modified phenotypes in the pairwise combinations (Table 1). We considered those most indicative of a true genetic interaction, where modified phenotypes occurred at least in three different KD/OE combinations and tissues and were markedly milder (antagonistic) or more severe or different than expected from adding the single phenotypes (synergistic) (Fig. 1c).

The most robust interaction was observed between *Ube3a* and *Mef2*. Combined knockdown in either wing (Fig. 2a,b) or eye (Fig. 3a,b) resulted in an amelioration of the phenotype observed in the single conditions. Wing venation defects as well as the rough eye with loss of ommatidia integrity, resulting in bubble-like appearance of the eye surface, improved. Pairwise overexpression of *Mef2* and *Ube3a* in either wing or eye led to a worsening of phenotypes. In the wing, the only mildly abnormal, curled wings in females from each of the single conditions were severely disorganized and not fully unfolded in the combined overexpression (Supplementary Fig. S1). While each of the single overexpression conditions was associated with a mild eye phenotype only, their combination resulted in a severe eye malformation with markedly reduced size and dissolved ommatidia structure (Fig. 3c,d). Overexpression of *Ube3a* and simultaneous knockdown of *Mef2* in either wing or eye also resulted in increased phenotypic severity, compared to the single conditions. Wings showed a severe disorganization in 75% of the females, which was not present in the single manipulations (Fig. 2c,d, Table 1). In the eye, the same combination resulted in partial male lethality and progressive necrotic patches in eyes of females (Fig. 3e,f, Tables 1 and S2). While pan-neuronal knockdown of *Mef2* did not affect viability and while pan-neuronal overexpression of Ube3a only resulted in partial lethality, a combination of these conditions resulted in complete lethality (Tables 1, S2). A similar observation was made for glial deregulation. There, simultaneous overexpression of both and simultaneous overexpression of *Mef2* with knockdown of *Ube3a* and vice versa resulted in complete lethality, while the single conditions were viable or only lethal in males (Tables 1, S2).

In summary, we have identified multiple phenotypic modifications upon pairwise dosage manipulation of *Ube3a* and *Mef2*. The synergistic or antagonistic direction of an interaction was dependent on the combination of KD and OE conditions and consistent across multiple tissues for each specific combination.

Also for combined dosage manipulation of *Ube3a* and *XNP*, phenotypic modifications were observed for several combinations and tissues. Simultaneous overexpression of *Ube3a* and knockdown of *XNP* in the wing resulted in increased viability of male flies (6% in OE_Ube3a alone and 39% in OE_Ube3a; KD_XNP, p < 0.001, Fisher’s exact test) and therefore in an improvement of the phenotype. In contrast, simultaneous knockdown of *Ube3a* and overexpression of *XNP* in the wing led to a more severe phenotype in the females, with stronger disorganization of cross veins compared to the phenotype in the single lines **(**Fig. 2e,f, Tables 1, S2**)**. Pan-neuronal overexpression of *Ube3a* in combination with knockdown of *XNP* resulted in worsening of incompletely penetrant lethality in females (20% of balancer flies in OE_Ube3a_1 alone and 10% in OE_Ube3a_1;KD_XNP, p < 0.05, Fisher’s exact test) and complete lethality in males (76% vs. 0%, compared to females, p < 0.05, and 23% vs. 0%, compared to balancer flies, p < 0.0001, Fisher’s exact test).

Pairwise manipulations of other tested genes did not result in multiple or consistent phenotypic modifications. When we evaluated the climbing assay upon pairwise dosage manipulations, we observed a significantly increased climbing impairment of the OE_*Da*;OE_*XNP* construct compared to the respective single conditions, which, however, might represent an additive effect of both single gene conditions. No other consistent modifications of the observed single phenotypes were observed **(**Supplementary Fig. S5**)**. Analysis of NMJ morphology and bang sensitivity upon pairwise dosage manipulations did not reveal any phenotypic modifications **(**Supplementary Fig. S4, Supplementary Table S3**)**.

### Protein co-localization and interaction

**Figure 4:**
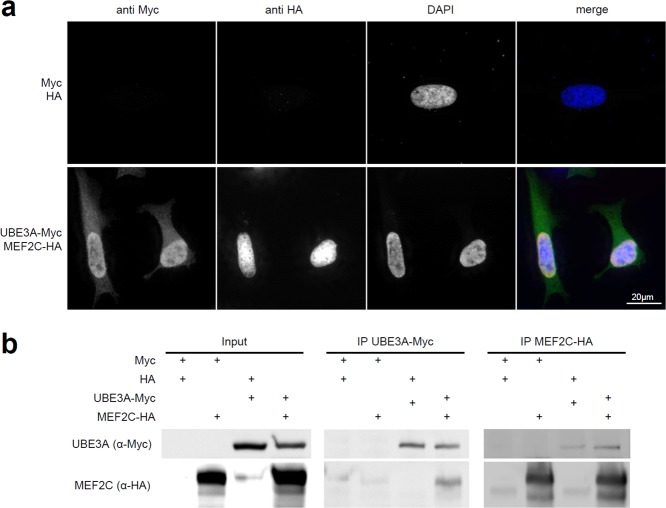
Physical interaction of human MEF2C and UBE3A in cells. (**a**) Co-localization studies of overexpressed human tagged MEF2C (HA, red) and UBE3A (Myc, green) in HeLa cells show diffuse nuclear localization of both proteins. (**b**) Co-immunoprecipitation experiments show physical interaction of overexpressed human MEF2C and UBE3A in HEK293 cells. Immunoprecipitation of Myc-tagged UBE3A also precipitates MEF2C-HA and vice versa. Please note, that the figure panels are cropped from two different blots with different exposure times. For full blots see Supplementary Fig. S6.

To investigate whether UBE3A and MEF2C also physically interact, we performed co-localization and co-immunoprecipitation studies in human cell lines. Firstly, we could confirm that UBE3A and MEF2C both localize to the nucleus as described previously^24^ when simultaneously overexpressed in HeLa cells, indicating that a physical interaction is possible (Fig. 4a). Subsequently, we could show that UBE3A and MEF2C can be co-immunoprecipitated from HEK293 cells co-transfected with Myc-tagged UBE3A and HA-tagged MEF2C. This was true for immunoprecipitation of either Myc-tagged UBE3A or HA-tagged MEF2C, suggesting that they indeed form a direct or indirect interaction (Figs. 4b, S6). Using quantitative RT-PCR, we tested if UBE3A/Ube3a or MEF2C/Mef2 might regulate transcriptional levels of each other. We did not find evidence for transcriptional effects as in fly larvae *Mef2* levels were unaltered upon *Ube3a* knockdown and vice versa (Supplementary Fig. S1). Furthermore, also in human cells (HEK293) *MEF2C* expression levels were unaltered upon *UBE3A* knockdown using siRNAs (Supplementary Fig. S7).

## Discussion

Though it has increasingly been acknowledged that similar clinical neurodevelopmental phenotypes are caused by mutations in genes/proteins connected in common networks and processes, this has mostly been characterized for specific complexes or pathways (e.g^2,4^.) or on a more systematic, large scale but consequently less detailed^1,18^ level. We now investigated possible functional links between *TCF4*, *MEF2C*, *ZEB2, UBE3A* and *ATRX* which are all implicated in clinically overlapping, severe human neurodevelopmental disorders. To do this, we utilized *Drosophila melanogaster* as a model system to systematically investigate functional links between the orthologues of these genes/proteins (*Da*, *Mef2*, Zfh1, *Ube3a*, *XNP*) *in vivo*.

### *Drosophila* lethality and morphological phenotypes point to developmental and glial roles

Our initial screen for lethality and gross morphological phenotypes upon ubiquitous and tissue specific gene dosage manipulation in the fruit fly identified a large number of quantifiable lethality and morphological phenotypes utilizable for the subsequent genetic interaction experiments.

In general, a high frequency of lethality upon ubiquitous but also upon tissue specific dosage manipulation underlines both the dosage sensitivity of these genes and their role for developmental processes. This has been shown in mouse models before. Ubiquitous knockout of either *Zeb2*^25^, *Tcf4*^26^, *Mef2c*^27^ or *Atrx*^28^ resulted in early lethality, while *Ube3a* brain-specific maternal-deficient mice displayed neurological deficits^29^. Interestingly, not only the loss of the maternal *UBE3A* copy in Angelman syndrome but also duplication of *UBE3A* in terms of duplication 15q syndrome is associated with a neurodevelopmental and epilepsy phenotype^30^, thus demonstrating bi-directional dosage sensitivity. We observed more frequent and more severe phenotypes upon overexpression than upon knockdown. Though knockdown might be a more suitable model for loss-of-function mechanisms, it might not always be phenotypically penetrant, particularly with RNA interference approaches as these usually leave a residual expression level of 30–50%. Therefore, overexpression screens provide a valuable additional tool to investigate gene function^31–33^ and to generate quantifiable phenotypes.

Contrary to our expectations for NDD-relevant genes, we did not observe consistent synaptic or behavioural phenotypes upon pan-neuronal manipulation in the utilized assays. Although all of the investigated genes are associated with epilepsy in humans, neither pan-neuronal knockdown nor overexpression of any single condition or combination resulted in bang sensitivity, a *Drosophila* model for seizure susceptibility, where mechanical shock can induce hyperactivity, spasms and paralysis^34^. This might suggest that seizures associated with haploinsufficiency of these transcriptional regulators might be related to different pathomechanisms from seizure-associated ion channel dysfunction which is typically reflected in bang sensitivity in flies^35^. Interestingly, in accordance with our findings, a previous report did not observe bang sensitivity upon neuronal overexpression of *Ube3a* as a model for epilepsy-associated duplications 15q11.2, either, but instead upon glial overexpression concomitant with down regulation of an ion pump^36^. Correlating a glial role of *Ube3a* between *Drosophila* and vertebrates is, however, difficult due to a discordant imprinting status of *Ube3a* in *Drosophila* neurons^37^, and as in a mouse model, *Ube3a* has shown to be expressed in glia but not to be imprinted there^38^. However, a possible, so far underestimated glial role of *Ube3a* and other genes investigated in this study would be in accordance with our observations that glial overexpression of either *Zfh1*, *Da*, *XNP* or *Mef2* resulted in lethality while this was only the case for pan-neuronal overexpression of *Zfh1* and with reduced penetrance for pan-neuronal overexpression of *Ube3a*. Although a relevance of Zfh1/ZEB2, XNP/ATRX or Da for (mainly peripheral) glia development and maintenance has been reported or discussed^39–43^, it has not yet been characterized in detail. Transcriptome analysis on cell populations from mouse brain and on human brain tissues summarized in the Brain RNA-Seq database indicates expression of all five genes not only in neurons but also in astrocytes, oligodendrocytes and microglia, with a higher expression of *TCF4*, *UBE3A* and *ATRX* in fetal compared to mature astrocytes^44,45^. Our observations therefore might suggest not only a role of neuronal but also of glial defects that might contribute to the neurodevelopmental phenotypes in humans with mutations in either of these genes.

### Genetic interaction between Ube3a and Mef2

Though the phenotypic overlap between the neurodevelopmental disorders investigated in this study is widely appreciated and discussed in the literature^7,46^, corresponding experimental follow-up of possibly underlying molecular commonalities is largely lacking. According to the mouse brain atlas^47^, there is overlapping expression of orthologues of UBE3A, MEF2C, ATRX, ZEB2 and TCF4 in several neuronal subtypes including excitatory neurons of the cerebral cortex and various cell types of the hippocampus. This would be in line with molecular commonalities in the pathomechanisms of the associated NDDs. However, according to the STRING database^48^ (status July 2019), there is no experimental evidence on interaction between the human proteins so far, and available data is restricted to co-expression or interaction in other species. In our genetic interaction screen, we observed modification of phenotypes upon combination of several manipulated genes. Severe phenotypes such as severe and fully penetrant lethality upon *Zfh1* overexpression precluded further evaluation for genetic interaction. For all other combinations, we observed modified phenotypes which might point to a functional link between these proteins in terms of genetic interaction (Table 1). Often, only two tissues and/or combinations were involved. More evidence was there for genetic interaction between Ube3a and XNP with modified phenotypes in two tissues and three different combinations. Further experimental follow-up would be necessary to confirm these potential interactions.

The most stringent evidence for a functional interaction was detected between Ube3a and Mef2. Several lines of evidence point to a true genetic interaction: a) we observed modified phenotypes in several tissues and in various knockdown/overexpression combinations, b) additive effects only can be excluded as some phenotypes occurred only upon pairwise manipulation (e.g. neuronal lethality) or were different or much more severe than expected from both single conditions (e.g. eye phenotypes), and c) we observed both antagonistic (milder phenotypes in eyes and wings upon simultaneous knockdown of *Ube3a* and *Mef2*) and synergistic effects (more severe phenotypes upon all other combinations). Additionally, we confirmed a physical interaction between human UBE3A and MEF2C in an independent cell-based system. To our knowledge, this is the first evidence for a functional link between UBE3A/Ube3a and MEF2C/Mef2 that might contribute to the phenotypic overlap between Angelman syndrome and *MEF2C*-associated intellectual disability. Apart from that, variants in either *UBE3A* or *MEF2C* might also represent modifiers for the phenotypic expression/severity of the respective other condition as discussed for CNV models^21,22^.

Taking into account the diverse functional roles of UBE3A and MEF2C, there are different possibilities conceivable how their interaction or mutual regulation might work. Observations from the genetic interaction and expression studies might already provide some insights into the possible nature of these interactions. The *UBE3A* gene encodes a member of the large family of E3 ubiquitin ligase proteins, initially termed E6-associated protein (E6-AP), and contributing to protein homeostasis by being involved in tagging substrate proteins with ubiquitin which are then degraded in the proteasome^9^. MEF2C (myocyte enhancer factor 2) belongs to the subfamily of MADS (MCM1-agamous-deficiens-serum response factor) transcription factors, whose transcriptional activity relies on the recruitment of and cooperation with other transcription factors as well as on translational and posttranslational modifications^49^. MEF2C might therefore be a transcriptional regulator of *UBE3A* expression. This might be supported by the identification of MEF2 binding sites in the *Ube3a* promoter^50^. Vice versa, also for UBE3A a transcriptional co-activation function has been reported^51^. However, a transcriptional regulation mechanism appears unlikely as in flies expression levels of *Mef2* were unaltered upon *Ube3a* knockdown and vice versa, and as in a human cell line *UBE3A* knockdown did not change *MEF2C* expression levels. Additionally, stem cell-derived neurons modelling Angelman syndrome, did not show significant transcriptional changes of other genes (compared to 15q duplication neurons)^52^. As the most likely hypothesis, we suggest that UBE3A might regulate MEF2C activity and levels by ubiquitination, leading to subsequent degradation in the proteasome. UBE3A has been found to be located in the neuronal nuclei in discrete hotspots over euchromatin, thus well-positioned to regulate active genes^24^, and such a ubiquitin-ligase-dependent regulation has been reported for other transcriptions factors before in mouse models, e.g. for SOD1^53^. Interestingly, only very recently, the critical importance of the nuclear isoform of UBE3A for the Angelman syndrome phenotype was characterized. Mice lacking the nuclear isoform but not mice lacking the cytosolic isoform displayed all major behavioural phenotypes and synaptic deficits also seen upon complete UBE3A knockout in the previous Angelman syndrome mouse model^54^. The hypothesis of ubiquitin-ligase dependent regulation of a putative nuclear substrate such as MEF2C by UBE3A is also supported by our genetic interaction findings. Pairwise knockdown of *Ube3a* and *Mef2* in the fly resulted in antagonistic genetic interaction with ameliorated eye and wing phenotypes. This might be explained by knockdown of *Ube3a* leading to decreased ubiquitination and degradation of Mef2. Subsequently increased Mef2 levels might result in a partial compensation of the *Mef2* knockdown phenotype. All other combinations resulted in synergistic genetic interaction, i.e. more severe phenotypes. This would be in line with a) overexpression of *Ube3a* resulting in increased ubiquitination and degradation of Mef2 and thus in a further decrease of its already low knockdown levels and b) knockdown of *Ube3a* resulting in decreased ubiquitination and degradation of Mef2 and thus in increased Mef2 abundance even above its overexpression levels. The observed interaction of MEF2C and UBE3A in co-immunoprecipitation experiments supports such a link. However, specific experimental proof of UBE3A regulating MEF2C in an ubiquitin-dependent fashion is still required.

By using *Drosophila melanogaster* as a model to screen for genetic interactions and by subsequent co-immunoprecipitation in a human cell line, we identified a robust interaction between UBE3A/*Ube3a* and *MEF2C/Mef2*. These molecular commonalities might contribute to the clinically overlapping features of the associated disorders.

## Material and Methods

### *Drosophila* lines and conditions

*Drosophila* orthologues of *TCF4* (daughterless [CG5102]), *ZEB2* (*Zfh1* [CG1322]), *MEF2C* (*Mef2* [CG1429]), *ATRX* (*XNP* [CG4548]), and *UBE3A* (*Ube3a* [CG6190]) were identified with DIOPT^55^. Manipulation of *Da/TCF4* in *Drosophila* has been established as a specific model for PTHS previously^56^.

All RNAi lines and the respective control (VDRC no. 60000) were obtained from the Vienna Drosophila Resource Center (VDRC)^57^. GAL4-driver stocks and transgenic lines for overexpression of *Zfh1* and *XNP*, respectively, were obtained from the Bloomington *Drosophila* Stock Center. UAS-*Mef2* was obtained from the Zurich ORFeome Project (FlyORF)^31^, and the UAS-Da line was a gift from Pascal Heitzler (IBMP Strasbourg). For generation of the UAS-*Ube3a* lines, the gene was amplified from fly cDNA (forward: 5’-GTAAAGTGCGCAGATTTCAGC-3’, reverse: 5’-GGTATCAGTTCCAGATGACAGAC-3’) and cloned into a pUAST fly expression vector^58^. After verification of the sequence, the vector was sent to BestGene Inc. for the creation of a stable transgenic line. For a complete list of used *Drosophila* lines see Supplementary Table S1. All overexpression lines were isogenized to the VDRC 60000 control by backcrossing for at least seven generations. Double-transgenic fly lines were generated using a double balancer line (Kr/CyO;D/TM6C) (Supplementary Table S2). Overexpression or RNAi-mediated knockdown was induced with the UAS-Gal4 system^58^ and confirmed by quantitative RT-PCR (Supplementary Fig. S1). In the text and figures, RNAi-lines are referred to as KD_gene, and UAS-lines as OE_gene. Flies were maintained on standard food, containing cornmeal, sugar, agar and yeast at 25 °C and bred at 28 °C because of temperature sensitivity of the UAS-GAL4 system^59^.

### Lethality and morphology analysis

RNAi- and transgenic overexpression lines were crossed to five different driver lines: *GMR*-GAL4 (eye), *ms1096*-GAL4 (wing), *repo*-GAL4 (glia cells), *elav*-GAL4 (pan-neuronal) and *Actin*-GAL4 (II, ubiquitous). Resulting offspring were counted and analysed with a Carl Zeiss™ 2000C stereo microscope for gross morphological abnormalities. If the driver line contained a balancer chromosome, offspring with balancers were counted, too. In crosses with double OE/KD constructs retaining balancers, the expected ratio of balancer to non-balancer offspring may deviate from 50%. Wings and eyes were analysed per fly, and a phenotype was counted when it occurred in at least one wing or eye per fly, respectively. Wings were visually evaluated for parameters such as shape, degree of unfolding and wing vein structure. Eyes were visually evaluated for parameters such as ommatidial structure, bristles and gross size and shape. If applicable, p-values were determined using Fisher’s Exact test.

### Climbing and bang sensitivity assays

Climbing behaviour and bang sensitivity was performed as described elsewhere^60^ upon pan-neuronal manipulation (*elav*-GAL4). In brief, offspring were collected within 72 h of eclosion under CO_2_ anaesthesia in groups of ten (5 males, 5 females, at least 40 flies tested per genotype). After 24 h, flies were transfered to the testing vial, tapped to the bottom and filmed for 30 s while climbing up. Time until 70% of the flies had crossed line at 8.8 cm height was measured from the videos. If less than 70% of flies from a vial managed to cross the line within the videotaped interval, time was considered to be 30 s. P-values were determined using the Wilcoxon-Mann-Whitney test, and Bonferroni correction was applied for multiple testing. For testing bang sensitivity, flies were vortexed for 10 s and filmed while recovering. The fraction of flies within a vial displaying spasms 5 s after vortexing was determined from the videotapes.

### Analysis of neuromuscular junctions (NMJs) from L3 larvae

Analysis of type 1b neuromuscular junctions (NMJs) of muscle 4 was performed as previously described^60^. Male L3 non-GFP larvae upon pan-neuronal manipulation (*elav*-GAL4/CyO-GFP;*elav*-GAL4) were dissected in PBST, fixated in 4% paraformaldehyde and stained with nc82 and anti-discs large antibodies (both from the Developmental Studies Hybridoma Bank, Iowa City, IA). Secondary antibodies used were Alexa 488 labeled anti-mouse antibody and the Zenon™ Alexa Fluor™ 546 Mouse IgG_1_ Labeling Kit (ThermoFisher Scientific). Images were taken with a Zeiss Axio Imager Z2 microscope in z-stacks and analysed in ImageJ^61^. NMJ area and length, as well as the number of active zones, synaptic islands, branches, and synaptic boutons were determined from the image stacks. Per genotype, at least 11 NMJs from at least four independent larvae were analysed. P-Values were determined using the Wilcoxon-Mann-Whitney test, and Bonferroni correction was applied for multiple-testing. Upon *Mef2* overexpression, we observed an additional signal in cross-segmental neurons with Dlg staining (red channel). This signal was also present in the parental *Mef2* line, in another line from the same background, and was also present without Dlg staining (Supplementary Fig. S8). It therefore most likely represents a background signal from the RFP gene under control of the artificial 3xP3 promoter present in the Fly ORF lines.

### RNA samples

For RNA sampling from flies, whole larvae (~5), adult flies (~4), heads (~10) or larval brains (~40) were collected and frozen at −80 °C for at least one hour. Total RNA was isolated with the RNeasy Lipid Tissue Mini Kit (Qiagen) using TRIzol™ (ThermoFisher Scientific) instead of QIAzol and QIAshredder columns (Qiagen) for homogenization. DNAse digestion was performed on-column with the RNAse-free DNAse kit (Qiagen). Reverse transcription of RNA into cDNA was performed using the SuperScript™ II reverse transcriptase (ThermoFisher Scientific). RNA from HEK293 cells was isolated using the RNeasy Minikit. DNAse digestion and reverse transcription was performed as described above.

### Quantitative reverse transcriptase PCR (quantitative RT-PCR)

Expression analysis was performed using the ABsolute QPCR SYBR Green ROX Mix (ThermoFisher Scientific) and (whenever possible) exon spanning primers (sequences available on request) on a QuantStudio™ 12 K Flex System (Life Technologies). Reactions were performed in quadruplicates, and Ct values were normalized to those of the endogenous controls *Actin* or *Tubulin* for *Drosophila* experiments or *B2M* for experiments on human cells. Relative expression levels were obtained using the ∆∆Ct method with isogenic background lines (*Drosophila*) or cells transfected with scrambled siRNA as references. Results were confirmed in at least a second biological replicate.

### Immunofluorescence

Expression plasmids expressing human *MEF2C* and *UBE3A* and respective negative control plasmids were obtained from Sino Biologicals (MEF2C-HA: HG12320-CY, UBE3A-Myc:HG11130-CM, pCMV-Myc:CV014 and pCMV-HA:CV013) and used for transient transfection. Transfected HeLa cells were grown on poly-lysine coated coverslips, fixated with 4% paraformaldehyde in PBS for 10 minutes and stained with anti-Myc (M4439, Sigma-Aldrich) and anti-HA (H6908, Sigma-Aldrich) and with Alexa Fluor™ 488 goat anti–mouse and Alexa Fluor™ 488 donkey anti–rabbit antibodies (A11001 and A10040, Thermo Fisher). Nuclei were counterstained with DAPI (Serva). Images were taken with a Zeiss Axio Imager Z2 Apotome microscope with a 63x objective and analyzed in ImageJ.

### Co-Immunoprecipitation

HEK293 cells were transiently transfected with a combination of UBE3A-Myc and MEF2C-HA or the respective negative controls and harvested 48 h post transfection. Cells were scraped from the culture dish in lysis buffer (100 mM TRIS-HCl pH8, 150 mM NaCl, 1 mM EDTA, 1% Triton X-100). Immunoprecipitation was performed with Protein A Mag Sepharose bead suspension (GE Healthcare), incubated with the sample and anti-Myc or anti-HA antibodies (M4439 or H6908, Sigma-Aldrich) at 4 °C overnight. Subsequently, beads were washed in lysis buffer, and samples were eluted with 1x Lämmli buffer.

Proteins were separated in stain-free 4–20% Mini-PROTEAN® TGX™ Precast Protein Gels (Bio-Rad), blots were stained with anti-Myc and anti-HA antibodies and imaged on a ChemiDoc™ Touch Imaging System (Bio-Rad).

### siRNA knockdown

Two different siRNAs against *UBE3A* (Qiagen) were transiently transfected into HEK293 cells using jetPrime (Polyplus) according to the manufacturer’s instructions. 48 h post transfection, RNA for expression analysis (see description above), was harvested.

## Supplementary information

Supplementary Information.

Supplementary Table S2.

## Data Availability

The datasets generated and/or analyzed during the current study are available from the corresponding author on reasonable request.
