# Supplementary material for: Genetic interaction screen for severe neurodevelopmental disorders reveals a functional link between Ube3a and Mef2 in Drosophila melanogaster
Source: Sci Rep. 2020 Jan 27;10:1204. doi: 10.1038/s41598-020-58182-5 (PMC6985129; doi:10.1038/s41598-020-58182-5)
Supplement: Supplementary file 1 — Supplementary Information. [file 41598_2020_58182_MOESM1_ESM.pdf]

Supplementary Data

Genetic Interaction screen for severe neurodevelopmental disorders reveals a functional link between *Ube3a* and *Mef2* in *Drosophila melanogaster*

Jonas Straub[1#], Anne Gregor[1#], Tatjana Sauerer[1], Anna Fliedner[1], Laila Distel[1], Christine Suchy[1], Arif B. Ekici[1], Fulvia Ferrazzi[1], Christiane Zweier[1*]

[1]Institute of Human Genetics, Friedrich-Alexander-Universität Erlangen-Nürnberg (FAU), 91054 Erlangen, Germany

[#] These authors should be regarded as joint first authors.

[*] To whom correspondence should be addressed: Christiane Zweier, phone: +49 9131 85 22319, fax: +49 9131 8523232, email: christiane.zweier@uk-erlangen.de

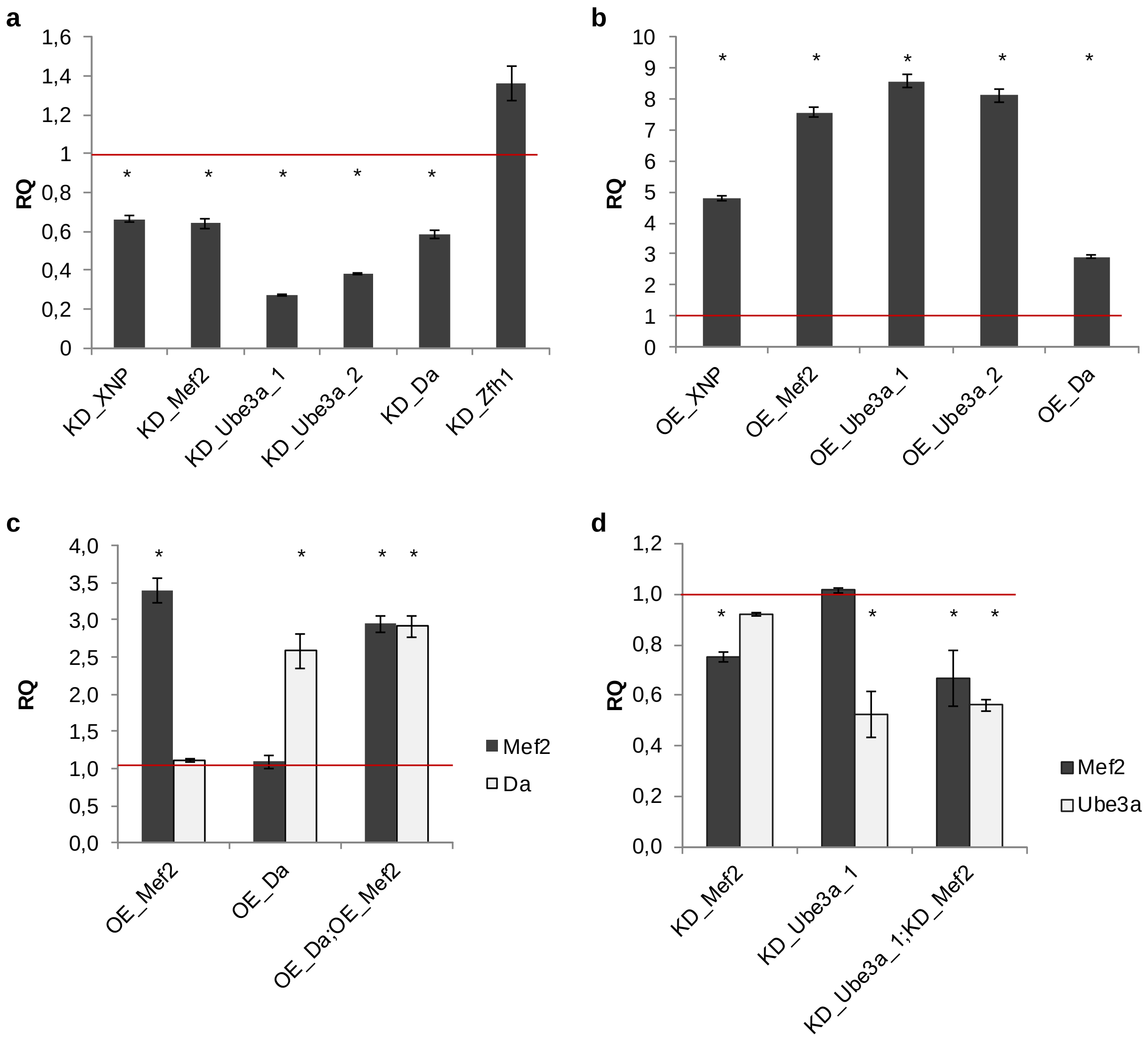

Supplementary Figure S1 Validation of *Drosophila* knockdown and overexpression. (**a**) Normalized results of quantitative RT-PCR after ubiquitous (*Actin*-GAL4 (II) driver: *XNP*, *Ube3a*_1/2: whole fly; *Actin*-GAL4 (III) *Mef2*, *Da*: larval brain) or pan-neuronal (*elav*-GAL4: *Zfh1*, heads) knockdown, confirming reduction of target-gene mRNA levels by ~35-70% for all used lines apart from KD_*Zfh1* which was excluded from further experiments. (**b**) Overexpression was confirmed for all used lines as ~3 to 8.5fold by quantitative RT-PCR on RNA from heads upon pan-neuronal (*elav*-GAL4) overexpression. Quantitative RT-PCRs for all lines apart from OE_*Da* was performed prior to isogenisation. (**c,d**) Quantitative RT-PCR upon overexpression (*elav*-GAL4, heads) or knockdown (*Actin*-GAL4 (III), whole larva) did not indicate reduced overexpression or knockdown strength for the double constructs compared to the single constructs. Reduced overexpression or knockdown efficiency by distribution of GAL4 on two different UAS-elements is therefore unlikely. Red lines indicate expression levels of respective

controls, bars represent mean normalized RQ values from four technical replicates in (**a-c**). Results were confirmed in an independent biological replicate (data not shown). Bars in (**d**) represent results from three biological replicates. *: $p < 0.01$ compared to control values (indicated by red line) (Wilcoxon rank sum test, tested on four technical replicates in (**a-c**) and three biological replicates in (**d**)). (**d**) mRNA levels of *Mef2* are unchanged upon knockdown of *Ube3a,* and mRNA levels of *Ube3a* are unchanged upon knockdown of *Mef2*, arguing against transcriptional regulation of Mef2 by Ube3a and vice versa.

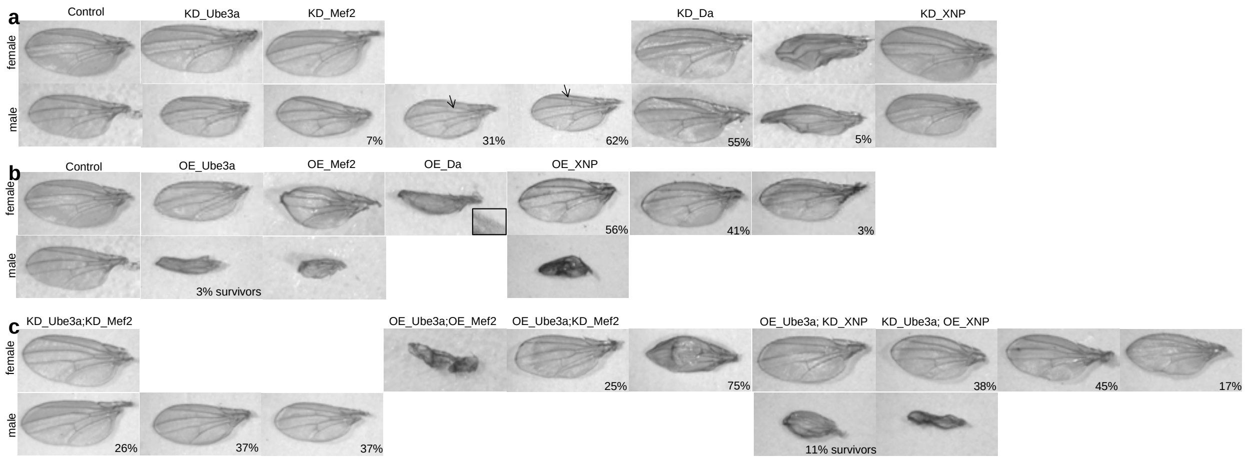

Supplementary Figure S2 Wing phenotypes. (**a**) Wing phenotypes upon wing-specific knockdown (*ms1096-GAL4*) of each gene. KD of *Ube3a* or *XNP* does not result in a phenotype, KD of *Mef2* leads to abnormal curling and variably disturbed cross vein architecture in males (7% both cross veins present, 31% anterior cross vein missing, 62% ectopic cross vein). KD of *Da* leads to a variable phenotype of normal, mildly affected (few bends or folds) or severely affected wings with severely impaired unfolding in females and males (55% mild, 5% severe). (**b**) Wing phenotypes upon wing-specific overexpression (*ms1096-GAL4*) of each gene. OE of *Ube3a* leads to almost complete male lethality with severely crippled wings (3% survivors), while female wings are abnormally curled. OE of *Mef2* leads to incomplete wing formation, while OE of *Da* is lethal in males and results in very small and curled wings without proper vein structure and with ectopic hairs in viable females. OE of *XNP* results in abnormally curled wings with varying degrees of cross vein architecture abnormalities in females (56% both cross veins present, 41% anterior cross vein lacking, 3% ectopic cross vein), while male wings show severe unfolding defects. (**c**) Simultaneous KD of *Ube3a* and *Mef2* results in a milder phenotype with significantly more male flies with both cross veins present (26%) and fewer male flies with ectopic cross veins (37%) (see also **Fig. 2**). Combined overexpression of *Ube3a* and *Mef2* resulted in more severely disorganized, unfolded wings in females and lethality in males. Simultaneous OE of *Ube3a* and KD of *Mef2* results in male lethality and in a more severe disorganization of wing architecture in about 75% of females (see also **Fig. 2**). Simultaneous KD of *Ube3a* and OE of *XNP* resulted in a more severe phenotype with more flies with ectopic cross veins and fewer flies with both cross veins intact (see also **Fig. 2**). Flies are counted towards the more severe phenotype if at least one wing was affected. These results are from independent experiments than in **Supplementary Table S2**, thus numbers are different.

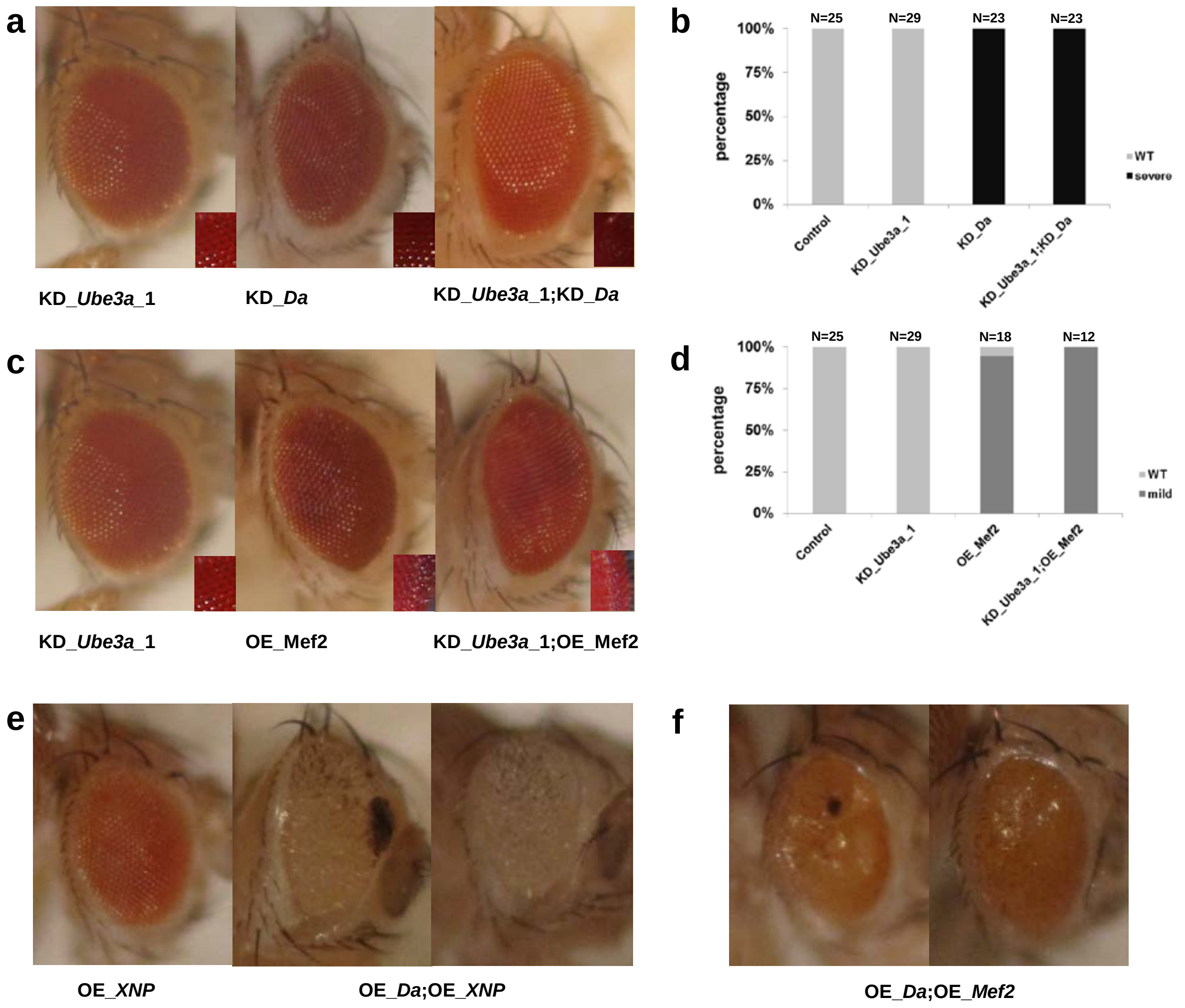

Supplementary Figure S3 Eye phenotypes. (**a**) Eye-specific KD (*GMR*-GAL4) of *Da* leads to a severe reduction of bristles (< 50% of wild type level) not observed for KD of *Ube3a*. Flies with KD of both, *Da* and *Ube3a* have the same phenotype as with KD of *Da* alone. (**b**) Quantification of **a**. (**c**) OE of *Mef2* in the eye causes a mild reduction of bristles (98-50% of wildtype (WT) level). No difference is observed for simultaneous KD of *Ube3a* and OE of *Mef2*. (**d**) Quantification of (**c**). (**e**) Eye-specific overexpression of *Da* is lethal (**Table 1, Supplementary Table S2**), while OE of *XNP* does not result in a gross eye phenotype. Simultaneous overexpression of *XNP* together with *Da* partially rescues the lethality with few female survivors displaying a severe eye phenotype with rough eye, stubby bristles and necrosis. (**f**) Few female survivors upon combined overexpression of *Da* and *Mef2* display a severe eye phenotype with rough eye, dissolved ommatidia structure and necrosis. All pictures apart from (**e**) and (**f**) are from male flies. Quantifications are from male flies.

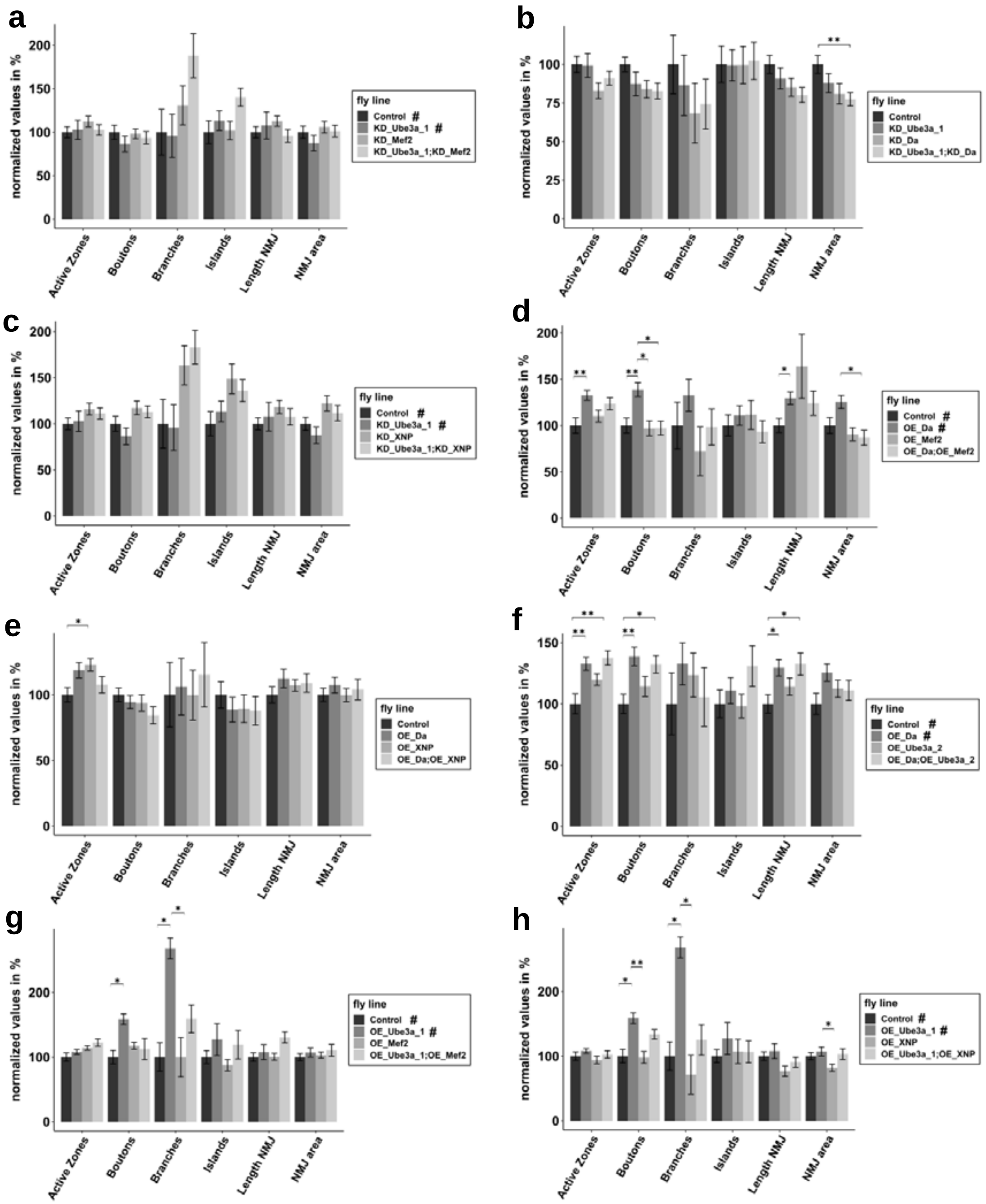

Supplementary Figure S4 Neuromuscular Junction (NMJ) morphology. Pan-neuronal knockdown (KD, **a-c**) or overexpression (OE, **d-g**) of the tested genes using the *elav*-GAL4 driver line did not result in significant or consistent/reproducible alterations in NMJ morphology regarding the assessed parameters. The diagrams represent the mean of a minimum of 11 NMJs from a minimum of four different larvae. All values are shown as % of the corresponding background control, error bars depict SEM. Asterisks indicate significance as determined by

Wilcoxon-Mann-Whitney test, Bonferroni correction was applied for multiple-testing (*: $p \leq 0.05$, **: $p \leq 0.01$). #: same results are also part of another diagram from the same experimental block.

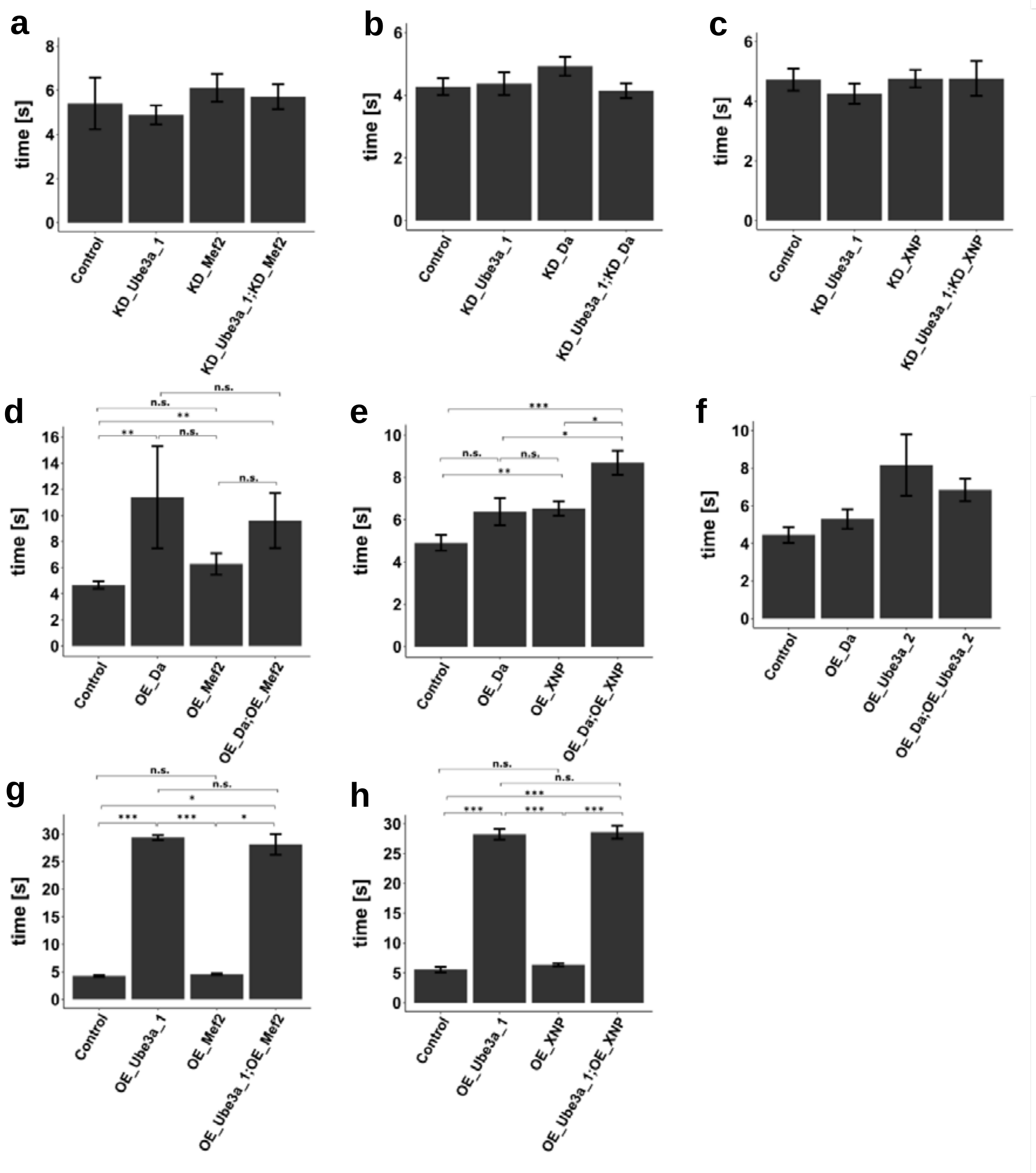

Supplementary Figure S5 Climbing assay. Upon pan-neuronal (*elav*-GAL4) knockdown (KD, **a-c**) or overexpression (OE, **d-g**), time was measured, until 7/10 flies needed to climb 8.8 cm after being tapped down in a vial. At least 40 flies were tested per genotype. OE of *Da* in one experiment and OE of *Ube3a* in two independent experiments resulted in significantly impaired climbing behavior compared to the isogenic control line. Double construct lines behaved in the

way of the more severe affected single construct they carried, apart from (**e**) where the double construct performed worse than each of the two single constructs. Bars represent mean values, error bars depict SEM, asterisks indicate significance as determined by Wilcoxon-Mann-Whitney test, Bonferroni correction was applied for multiple-testing (n.s.: not significant, *: $p \leq 0.05$, **: $p \leq 0.01$, ***: $p \leq 0.001$). For (**a-c**) and (**f**) all comparisons were non-significant. Note that for (**a**), flies were tested 48h post collection and 24h after having been tested for bang sensitivity.

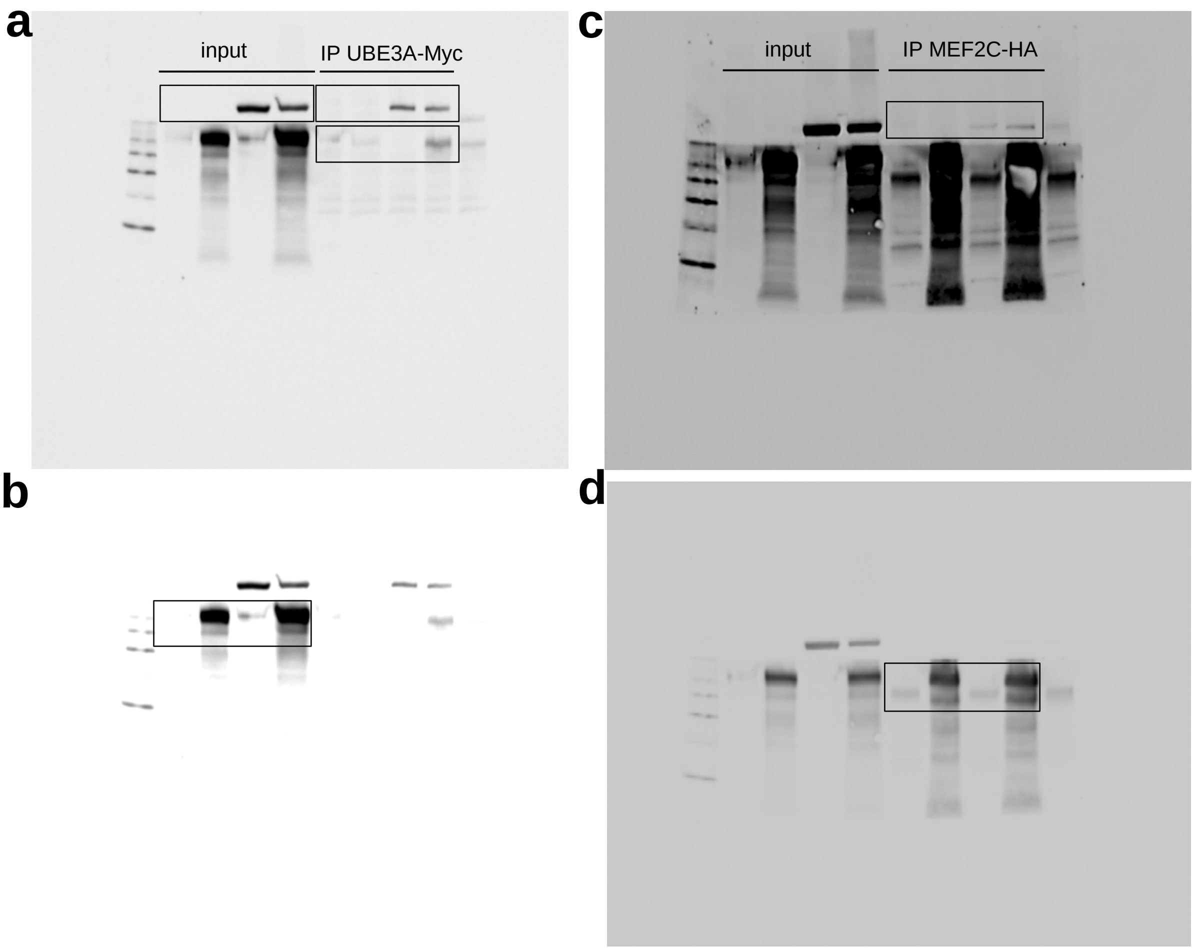

Supplementary Figure S6 (**a,b**) Identical full blot with two different exposure times. Boxes indicate the cropped cut-outs displayed as the first four panels of **Fig. 4b**. (**c,d**) Identical full blot with two different exposure times. Boxes indicate the cropped cut-outs displayed as the last two panels of **Fig. 4b**.

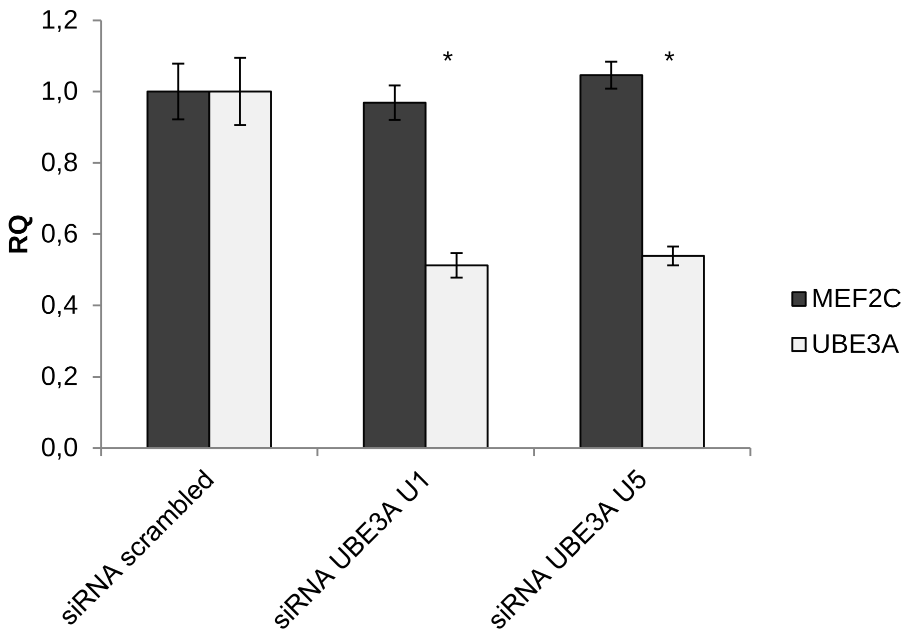

Supplementary Figure S7 Expression analysis of *MEF2C* in *UBE3A* knockdown cells. HEK293 cells were transfected with two different siRNAs against UBE3A (U1 and U5) or a scrambled siRNA control. Normalized results showed knockdown of *UBE3A* to 50% of control levels, while *MEF2C* expression remained unchanged. Bars represent mean normalized RQ values from three biological replicates with standard deviation. *: $p < 0.01$ compared to control values (Wilcoxon rank sum test).

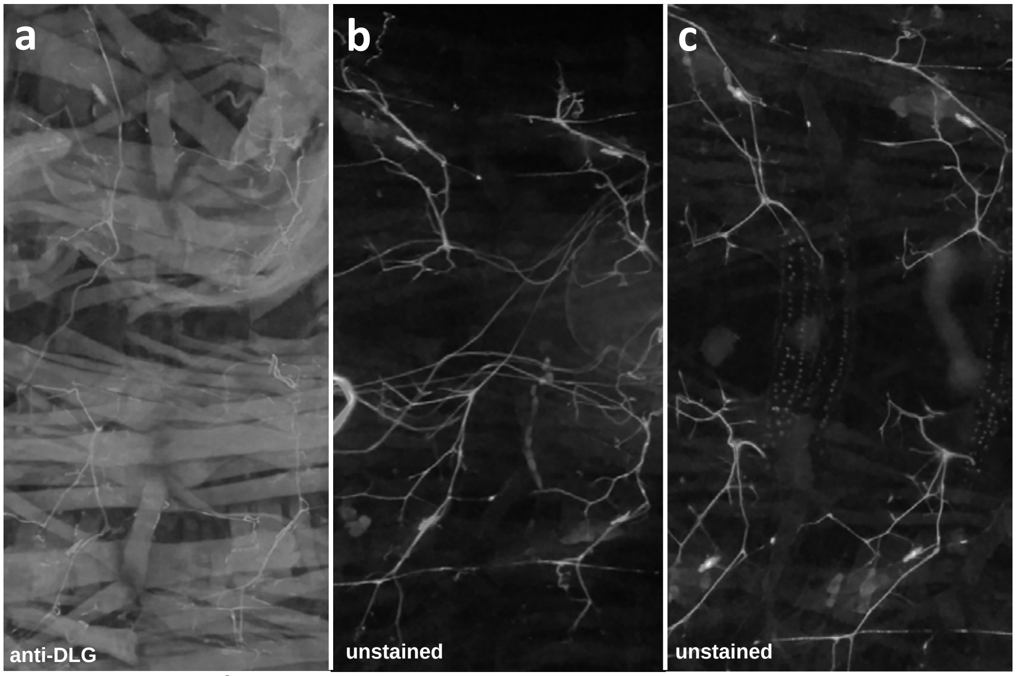

pan-neuronal OE_*Da*;OE_*Mef2* | **F000030 (Mef2) (un-crossed line)** | **F000619 (Ctcf) (un-crossed line)**

Supplementary Figure S8 Potential background activity of the 3xP3 promoter present in FlyORF lines. (**a**) When analyzing NMJs upon pan-neuronal (*elav*-GAL4;*elav*-GAL4) *Mef2* overexpression (FlyORF line F000030, isogenized), we identified a strong fluorescence signal in the red DLG-stained channel resembling motoneurons. (**b**) The same signal was detected in the unstained parental line, not crossed to the *elav*-GAL4;*elav*-GAL4 promoter and (**c**) in another, unrelated line from FlyORF (Ctcf, F000619).

FlyORF transgenic lines carry the *RFP* gene under control of the artificial 3xP3 promoter[1]. The 3xP3 promoter is known to be active in the *Drosophila* eye[2]. We therefore assume that the "motoneuron" signal stems from a yet undescribed activity of the artificial 3xP3 promoter in the developing *Drosophila* nervous system.

Supplementary Table S1 Used *Drosophila* lines.

**Name**	**Line**	**Origin**
actin-GAL4 (II)	y[1] w[*]; P{w[+mC]=Act5C-GAL4}25FO1/CyO, y[+]	BL#4414
actin-GAL4 (III)		
repo-GAL4	w[1118]; P{w[+m*]=GAL4}repo/TM3, Sb[1]	BL#7415
elav-GAL4	P{w[+mC]=GAL4-elav.L}2/CyO	BL#8765
elav-GAL4;*elav*-GAL4	*elav-GAL4/CyO-GFP;elav-GAL4*	Gift Annette Schenck, Nijmegen
GMR-GAL4	w[*]; P{w[+mC]=GAL4-ninaE.GMR}12	BL#1104
ms1096-GAL4	w[1118] P{w[+mW.hs]=GawB}Bx[MS1096]	BL#8860
Kr/CyO;D/TM6C		gift Annette Schenck, Nijmegen
KD_*Ube3a_1*	UAS-RNAi-*Ube3a*	VDRC 45876/GD
KD_*Ube3a_2*	UAS-RNAi-*Ube3a*	VDRC 45875/GD
KD_*XNP*	UAS-RNAi-*XNP*	VDRC 10618/GD
KD_*Mef2*	UAS-RNAi-*Mef2*	VDRC 15550/GD
KD_*Da*	UAS-RNAi-*Da*	VDRC 51297/GD
KD_*Zfh1*	UAS-RNAi-*Zfh1*	VDRC 103205/KK
OE_*Ube3a_1*	UAS-*Ube3a*	BestGene Inc., 16534-2-4M, isogenized at least 7 generations with VDRC 60000
OE_*Ube3a_2*	UAS-*Ube3a*	BestGene Inc., 16534-2-3M, isogenized at least 7 generations with VDRC 60000
OE_*XNP*	w[*]; P{w[+mC]=UAS-XNP.HA}3	BL#26645, isogenized at least 7 generations with VDRC 60000
OE_*Mef2*		FlyORF F000030, isogenized at least 7 generations with VDRC 60000
OE_*Da*	UAS-*Da*	gift from Pascal Heitzler, Strasbourg, isogenized at least 7 generations with VDRC 60000
OE_*Zfh1*	w[1118]; P{w[+mC]=UAS-zfh1.P}2B	BL#6879, isogenized at least 7 generations with VDRC 60000
control	w[1118]	VDRC 60000
control KK	y,w[1118];P{attP,y[+],w[3`]	VDRC 60100

Supplementary Table S2 Lethality and morphology. (excel file)

Supplementary Table S3 Results of bang sensitivity assay. Fraction of 10 flies displaying spasms 5s after vortexing after pan-neuronal knockdown or overexpression (*elav*-GAL4). In all cases less than 25% of flies displayed spasms at the selected time and we therefore consider them to be bang sensitivity negative. Experiments were performed in blocks of four (control, Line A, Line B, Line A;B).

Condition (pan-neur.)	fraction	SEM
**Control**	0.091666667	0.02599048
**KD_Ube3a_1**	0.066666667	0.02357023
**KD_Mef2**	0.058333333	0.02289083
**KD_Ube3a_1;KD_Mef2**	0.175	0.04118772
**Control**	0.023529412	0.01363767
**KD_Ube3a_1**	0.076923077	0.02307692
**KD_Da**	0.1	0.02415229
**KD_Ube3a_1;KD_Da**	0.13125	0.03125
**Control**	0.1	0.02567763
**KD_Ube3a**	0.1	0.02581989
**KD_XNP**	0.08125	0.02085416
**KD_Ube3a;KD_XNP**	0.158823529	0.03327561
**Control**	0.15	0.03743126
**OE_Da**	0.028571429	0.02857143
**OE_Mef2**	0.077777778	0.0190554
**OE_Da;OE_Mef2**	0.04	0.0305505
**Control**	0.047058824	0.01740023
**OE_Da**	0.03	0.01791794
**OE_XNP**	0.00625	0.00625
**OE_Da;OE_XNP**	0.011111111	0.00762216
**Control**	0.134307143	0.08355331
**OE_Da**	0.06	0.03399346
**OE_Ube3a_2**	0.205555556	0.03075544
**OE_Da;OE_Ube3a_2**	0.085714286	0.04592215
**Control**	0.068421053	0.02030849
**OE_Ube3a_1**	0.1	0.04605662
**OE_XNP**	0.021052632	0.00960917
**OE_Ube3a_1;OE_XNP**	0.192307692	0.03092907
**Control**	0.05625	0.01818596
**OE_Ube3a_1**	0.109090909	0.02845905
**OE_Mef2**	0.063157895	0.01745592
**OE_Ube3a_1;OE_Mef2**	0.1	0.04472136

Supplementary References

1 Bischof, J., Sheils, E. M., Bjorklund, M. & Basler, K. Generation of a transgenic ORFeome library in Drosophila. *Nat Protoc* **9**, 1607-1620, doi:10.1038/nprot.2014.105 (2014).

2 Horn, C. & Wimmer, E. A. A versatile vector set for animal transgenesis. *Dev Genes Evol* **210**, 630-637 (2000).
